# Proximal humeral multiple fragment fractures in patients over 55: Comparison between Conservative treatment and Plate Fixation

**DOI:** 10.1016/j.heliyon.2024.e25898

**Published:** 2024-02-10

**Authors:** Marco Sapienza, Vito Pavone, Liliana Muscarà, Pierpaolo Panebianco, Alessia Caldaci, Kathryn Louise McCracken, Giuseppe Condorelli, Vincenzo Fabrizio Caruso, Danilo Costa, Angelo Di Giunta, Gianluca Testa

**Affiliations:** a Department of General Surgery and Medical Surgical Specialties, Section of Orthopaedics and Traumatology, P.O. “Policlinico Gaspare Rodolico”, University of Catania, Catania, Italy; b School of Medicine, University College Cork, College Road, T12 K8AF, Cork, Ireland; c Orthopaedic Division of Policlinico “G.B. Morgagni”, 95125, Catania, Italy

**Keywords:** Humerus proximal fracture, Trauma, Surgical technique, Conservative treatment, Comparison

## Abstract

Proximal humerus fractures account for about 5% of fragility fractures. These involve a significant burden of disability and a reduced quality of life. This study aims to compare functional results and surgical outcomes (closed reduction and internal fixation with the internal closure system of the proximal humerus) and the conservative management of proximal humerus fractures by 2-, 3-, 4-parts, in patients older than 55 years. Between January 2017 and April 2019, 65 patients with 2, 3 or 4-part fractures were retrospectively analyzed: 29 patients (5 males and 24 females) with an average age of 70.8 ± 9.9 years treated non-surgically (conservative group (CG)) and 36 patients (11 males and 25 females) with an average age of 66.2 ± 7.1 years treated surgically with plate fixation (operating group (OG)). Using different evaluation scores, we compared the OG and the CG. Through the DASH score we have seen how at 12 months there is a satisfactory result in patients with conservative treatment (p = 0.0019). Constant-Murley scale shows no difference between the two treatments (p = 0.2300). BARTHEL scale and SST score did not give statistically satisfactory results. Also, after one year of follow-up, patients treated with conservative therapy had a higher improvement in their Range of Motion (ROM) values than patients treated with surgical treatment. The results in terms of pain in NPRS at 3, 6, 12 months are better for conservative groups (p = 0,0000). Our findings suggest that conservative treatment in proximal humeral fractures, particularly in multi-fragmented fractures in patients over 55 years of age, designs an excellent alternative to the surgical option.

## 1 Introduction

Proximal humerus fractures are the third most common fragility fracture (about 5%) and they are associated with a substantial burden of disability and impaired quality of life [1]. Due to the increased life expectancy and the resulting ageing of the population, the incidence and frequency of these fractures is increasing. Most of them take place after the age of 50 [2]. In relation to frequency Roux et al. [3], report that the critical age for this type of trauma is 70 years and affects the dominant arm in 48% of cases. The main cause of this type of fracture in men is in 55% of cases a simple fall and in the remaining 45% a high energy trauma. In women, on the other hand, the percentage of fractures caused by simple falls rises to 82% of cases [3]. The risk factors predisposing to this traumatic event are two: bone fragility (related to the increase in osteoporotic state in women in the postmenopausal period) and the and high energy traumas, especially in the young population [3,4]. Traumatic mechanisms necessary to produce a proximal humerus fracture may be caused by direct trauma or because of an axial load transmitted to the humerus through the elbow or through the hand and forearm posed in extension or with the elbow blocked in extension [5].

The 20% of proximal humerus fractures requiring surgical treatment. Over the years, several methods have been proposed for proximal humeral fractures treatment but there is currently no universally recognized protocol [6]. The choice of an appropriate treatment depends on the type of fracture, bone quality, deforming forces, surgeon skills, compliance, and expectations of the patient. Most of proximal humeral fractures are undisplaced or minimally displaced and they can be treated conservatively through a period of immobilization with a brace and subsequent cautious mobilization. In 10–20% of cases, fractures can present a significant displaced and a surgical treatment is required. Surgery in combination with proper rehabilitation plays a key role in the patient's recovery activity. The aim of the surgery is to obtain a good bone fragments reduction in order to allow an early rehabilitation as soon as possible. Multiple treatment methods have been used in the surgical management of these fractures including proximal humeral nails (PHN), percutaneous K-wiring (PKW), percutaneous fixed angle locking plates (LP), open reduction and internal fixation with LP and partial or total arthroplasty. So, it becomes necessary to define valid criteria for the selection of patients with certain types of fractures to identify the appropriateness of the type of treatment [[7], [8], [9]]. Nowaday, the treatment of proximal humerus fractures, available therapeutic options and decision-making are still debated. Each type of surgery has advantages and disadvantages, and the treatment should be individualized according to a list of factors including patient characteristics (age, health status, dominant limb, gender, habitus, lifestyle), biomechanical characteristics of the joint (fracture morphology, trabecular and cortical bone quality, cuff function, vascular system integrity) and surgeon preferences. [[5], [6], [7], [8], [9], [10]].

The purpose of this study was to compare the functional results and the outcomes of operative (closed reduction and internal fixation with the proximal humerus internal locking system) and nonoperative management of proximal humerus fractures in patients older than 55 years. Our hypothesis was that the clinical outcomes of these 2 treatment methods should be similar.

## 2 Materials and methods

### 2.1 Declarations

Informed consent was obtained from all patients for the publication of all their data and/images.

This prospective cross-sectional study was conducted according to the guidelines of the Declaration of Helsinki and approved by the Institutional Review Board A.O.U. Polyclinic “G. Rodolico -San Marco” of Catania (protocol 117/2020/PO, 14 October 2020).

Between January 2017 and April 2019, 65 patients have been assessed retrospectively with 2-, 3-, or 4- part fractures, with 29 patients treated non-operatively (Conservative group - CG) with an average age of 70.8 ± 9.9 years, and 36 treated surgically (Operative group - OG) with an average age of 66.2 ± 7.1 years. These patients were selected and analyzed retrospectively. Inclusion criteria were: 1) patients over 55 years aged; 2) displaced proximal humerus fracture (Neer's criteria were used to define displacement: fractures displaced 1 cm or more and/or with angulation of 45° or more); 3) minimum follow-up of one year; 4) closed reduction and internal fixation with the proximal humerus internal locking system (OG). Exclusion criteria were: 1) patients presented more than 1 week after injury; 2) refractures; 3) non-union, infection or pathologic fracture; 4) if there was an associated neurovascular injury; 5) if the injury radiographs were absent or inadequate; 6) previous surgery; 7) neuromuscular disease; 8) coexisting fractures of the ipsilateral extremity; 9) if a fixation method other than locked plating was used.

### 2.2 Demographic data

To get two comparable patient groups, the patients from CG were directly matched with patients from the OG. Demographic and clinical data were recorded (Table 1), in the presence of the patient, as follows: patient age and sex, mechanism of injury, side of injury, any associated orthopaedic injuries, number of days between injury and presentation. The first radiographs and any CT scan presented were studied and each fracture type was assessed according to Neer classification [11] and Hertel classification (LEGO) [12].

**Table 1:** Anthropometric characteristics of the Study Cohort.

	Total cohort	CG	OG	p-value
Number of patients	65	29	36	
Average age	68.5 ± 3.2	70.8 ± 9.9	66.2 ± 7.1	<0.05
Gender	21 males 44 females	5 males 24 females	11 males 25 females	

Abbreviations: CG: conservative Group; OG: operative group.

For each patient have been assessed: age, sex, date of trauma and possible subsequent surgery, and the mechanism of the trauma. Investigations were partially conducted by telephone due to the lockdown period caused by the Covid-19 pandemic and the consequent impossibility to be calling patients to the clinic for the period March–May 2020.

### 2.3 Management

Both groups were treated by two senior shoulder surgeons, clinical data were collected from the databases of the two centres and from the analysis of the clinical records and certificates issued to patients. The CG's patients were treated at the Polyclinic “G.B. Morgagni” Mediterranean Foundation, Orthopedics Traumatology and Rehabilitation Unit, (Catania, Italy). The shoulder was immobilized in a sling for 5 weeks with the arm in adduction and internal rotation, and flexed elbow. During these weeks, the patient was also advised not to place the elbow of the affected limb on chairs with armrests and to sleep in a supine position with a pillow positioned behind the arm. The passive and active ROM exercises starting after 5 weeks and were referred to standardized intensive physical therapy and rehabilitation. After 45 days and 12 weeks, patients were reviewed in the outpatient department. Starting from 12 weeks they were then seen 3 and 6 months later. X-ray images were made at each of these visits to assess complications.

The OG's patients were treated at Orthopedics and Traumatology Unit, A.O.U. Policlinico-San Marco, University of Catania (Catania, Italy). All surgeries were performed in the beach chair position, with a high anterior-lateral deltoid split incision. The fractures were reduced under x-ray image intensifier and in all operations, non-contact-bridging - NCB ® (Plate for the proximal humerus - PH - Zimmer, Inc.; Warsaw, Indiana, USA) plate were used. After surgery, immobilization was performed with a sling, with the arm in adduction and internal rotation, and flexed elbow. Patients without any problems were discharged 3 days postoperatively and sutures were removed after 15 days. Passive and active ROM exercises were started at 2–4 weeks postoperatively relying on the stability of the osteosynthesis and quality of bone. Then patients were referred to physical therapy and rehabilitation. After 2, 4 and 12 weeks, patients were examined in the outpatient ward and X-ray images were taken at each of these visits.

### 2.4 Outcome measures

So, to determine clinical outcomes, patients were interviewed in an outpatient setting. Three standardized questionnaires were completed to review the patients’ shoulder function and general health state at 1-3-6-12 months after treatment: the Constant-Murley [13], the Disabilities of the Arm, Shoulder, and Hand (DASH) [14], and Simple Shoulder Test (SST) [15]. For the general health state of the patient, the Barthel Index was used. A Numeric Pain Rating Scale (NPRS) [16] was used to grade patient satisfaction.

The shoulder active ROM of all patients were assessed using a universal long-arm goniometer to measure flexion, extension, abduction, external rotation, and internal rotation. The ROM of the affected side was compared with the unaffected side.

### 2.5 Statistical analysis

Continuous data are presented as means and standard deviations, as appropriate. The analysis of variance test with *t*-test and Tukey–Kramer method were used to compare the clinical assessment. The selected threshold for statistical significance was p < 0.05. All statistical analyses were performed using the 2016 GraphPad Software (GraphPad Inc, San Diego, California).

## 3 Results

In our case history a total of 65 patients with an average age of 68.5 ± 3.2 were evaluated. Divided into two study groups: the CG treated with conservative treatment was composed of 29 patients (5 males, 24 females) with an average age of 70.8 ± 9.9 years. The OG treated with plate fixation was composed of 36 patients (11 males, 25 females) with an average age of 66.2 ± 7.1 years.

**Fig. 1:**
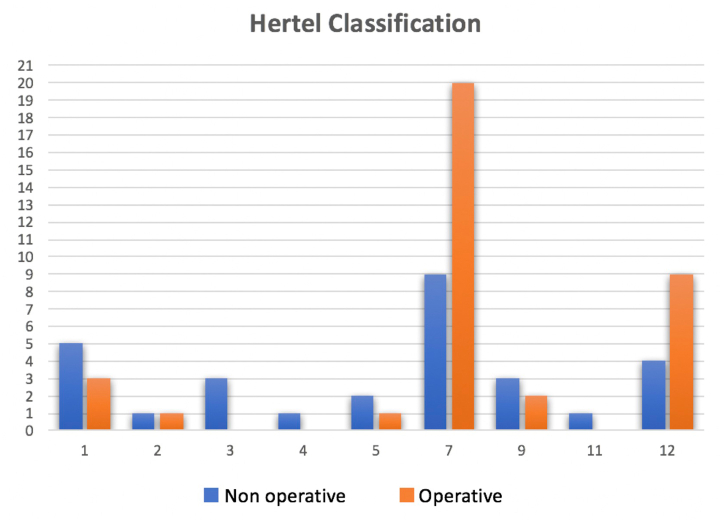
Hertel Classification of patients of both groups: Non-Operative and Operative.

Fractures in both groups were evaluated with Hertel's classification: in the first group 5 patients were classified as type 1; 1 patient type 2; 3 patients type 3; 1 patient type 4; 2 patients type 5; 9 patients type 7; 3 patients type 9; 1 patient type 11; and finally, 4 patients type 12. In the second group 3 patients were classified with fracture type 1 according to Hertel; 1 patient type 2; 1 patient type 5; 20 patients type 7; 2 patients type 9, and 9 patients type 12. (Fig. 1).

There are differences between the two groups in terms of DASH score. There is a more rapid improvement in operated patients than conservative treated patients from the third to the sixth month post-operative period, but it isn't statistically significant (p = 0,0520). Instead, there is a statistically significant difference between functional result at 12 months in favor of conservative treated patient (p = 0,0019). (Tabel 2).

The scores obtained with the Constant-Murley scale have no significant differences between the two groups (p = 0.2300) (Tabel 3).

At 12 months of follow up the relative Constant-Murley's score were on average of 95.2% ± 12.9 for the conservative treatment, instead of patients in the plate fixation group which presented a Constant-Murley's age related score with an average value of 83.2% ± 18.6.

The BARTHEL scale shows less level of disability for non-operative group in the 12° month, but this conclusion is not statistically significant (p = 0,1162) and this result was reached slower than the other group. (Tabel 4).

The SST score shows a better functional outcome for conservative group in the 12° month, but this conclusion is not statistically significant. (Table 5).

DASH, CMS, SST, BARTHEL scores and ROM improved significantly from 1° to 12° months after both conservative and operative treatment (P = 0.001).. (Table 2, Table 3, Table 4, Table 5, Table 6, Table 7, Table 8).

**Table 2:** According to 1-, 3-, 6- and 12-months follow-up DASH score results.

Group	DASH 1 month	DASH 3 month	DASH 6 month	DASH 12 month	*P value*
CG	63,2	39,9	23,0	11,3	0,0001
OG	65,3	47,2	34,5	29,1	0,0001
CG vs OG	0.9998	0.7182	0.1600	0.0019	

Abbreviations: CG: conservative Group; OG: operative group; DASH: Disabilities of the Arm, Shoulder and Hand.

**Table 3:** According to 1-, 3-, 6- and 12-months follow-up Constan-Murley score results.

Group	CMS 1 month	CMS 3 months	CMS 6 months	CMS 12 months	*P value*
CG	19,9	38,1	54,4	66,0	0.0001
OG	19,4	35,9	50,4	60,6	0.0001
CG vs OG	1.0000	0.9753	0.6190	0.2300	

Abbreviations: CG: conservative Group; OG: operative group; CMS: Constan-Murley score.

**Table 4:** According to 1-, 3-, 6- and 12-months follow-up Barthel score results.

Group	BARTHEL 1	BARTHEL 2	BARTHEL 3	BARTHEL 4	*P value*
CG	72,8	73,4	84,7	97,9	0.0001
OG	69,9	79,7	85,4	89,4	0.0001
CG vs OG	0.9824	0.4643	1.0000	0.1162	

Abbreviations: CG: conservative Group; OG: operative group.

**Table 5:** According to 1-, 3-, 6- and 12-months follow-up SST score results.

Group	SST 1 month	SST 3 months	SST 6 months	SST 12 months	*P value*
CG	26,7	44,5	60,0	73,6	0.0001
OG	24,1	45,6	56,5	60,6	0.0001
CG vs OG	0.9995	1.0000	0.9965	0.1424	

*Abbreviations: CG: conservative Group; OG: operative group; SST:* Simple Shoulder Test.

**Table 6:** According to 1-, 3-, 6- and 12-months follow-up NPRS score results.

Group	NPRS 1 month	NPRS 3 months	NPRS 6 months	NPRS 12 months	*P value*
CG	7,0	3,4	2,3	73,6	0.0001
OG	7,3	5,8	4,3	60,6	0.0001
CG vs OG	0.99	0.0000	0.0000	0.0000	

*Abbreviations: CG: conservative Group; OG: operative group; NPRS: Numerical Pain Rating Scale*.

**Table 7:** According to 1-, 3-, 6- and 12-months follow-up Flexion-Abduction ROM results.

Group	FLEXION				*p*	ABDUCTION				*p*
	1	3	6	12		1	3	6	12	
CG	19,2	107,9	135,2	153,4	0.0001	20,3	83,6	124,1	134,8	0.0001
OG	23,9	88,9	111,7	127,5	0.0001	23,9	72,8	83,1	104,4	0.0001
CG vs OG	0.9781	0.0088	0.0002	0.0000		0.9803	0.3025	0.0000	0.0000	

Abbreviations: CG: conservative Group; OG: operative group.

**Table 8:** According to 1-, 3-, 6- and 12-months follow-up External-Internal Rotation ROM results.

Group	ER				*p*	IR				*p*
	1	3	6	12		1	3	6	12	
CG	−3,5	24,8	135,2	153,4	0.0001	20,3	83,6	124,1	134,8	0.0001
OG	−1,1	23,6	111,7	127,5	0.0001	23,9	72,8	83,1	104,4	0.0001
OG vs CG	1.000	0.9903	0.1668	0.0000		0.9901	0.9901	0.0006	0.0000	

Abbreviations: CG: conservative Group; OG: operative group; ER: External-Internal Rotation; IR: Internal Rotation.

The results in terms of pain in NPRS at 3, 6, 12 months are better for conservative groups (p = 0,0000) but comparable to the other group in the 1° month (p = 0,99). (Table 5). The Range of Motion (ROM) in conservatively treated patients passed, after one year of follow-up, from functional impotence to mean values in flexion of 153.4° ± 14.7°, in abduction of 134.8° ± 16.2°, in extra rotation of 38.1° ± 5.6° and at an intra-rotation level of 63.1° ± 17.1°.

On the other hand, surgically treated patients also improved their average values after one year of follow-up, although less than conservatively treated patients: in flexion of 127.5.2° ± 15.5°, in abduction of 104.4° ± 17.2°, in extra rotation of 24.4° ± 14.3° and at an intra-rotation level of 41.9 ± 27.1°. (Tabel 6, tabel 7).

DASH, CMS, SST, BARTHEL scores and ROM improved significantly from 1° to 12° months after in both groups (P = 0.001). (Table 2, Table 3, Table 4, Table 5, Table 6, Table 7, Table 8). None of the patients reported complications in the 12 months of follow-up.

## 4 Discussion

Despite the high incidence and costs of proximal humerus fractures, there is currently no valid scientific evidence for the best treatment method [17]. Our study is a multidisciplinary analysis in which patients were managed by the same two surgeons, upper extremity specialist.

**Fig. 2:**
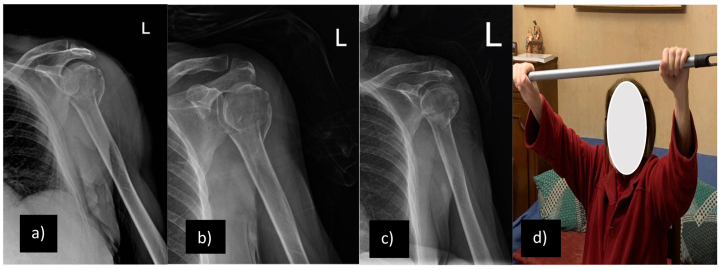
X-ray and clinical images of OG patient. a) at the time of trauma; b) 1 month from conservative treatment; c) 6 months from conservative treatment; d) clinical image after 3 months from conservative treatment and 2 months after physiotherapy treatment. Abbreviations: CG: Conservative Group.

The CG was treated by the team of specialists of the Polyclinic “G.B. Morgagni” Mediterranean Foundation, Orthopedics Traumatology and Rehabilitation Unit, (Catania, Italy), where criteria for the choice of treatment type include calcar size less than 8 mm, decomposition of the medial hinge greater than 2 mm, as described by Hertel. Moreover, concomitant decisional elements were the maintenance of the glenohumeral relationship after fracture, due to the risk of subsequent impingement; the presence of relevant comorbidities that contraindicate surgery; the functional demand of the patient (generally lower in elderly subjects). (Fig. 2 a-b-*c*- d).

**Fig. 3:**
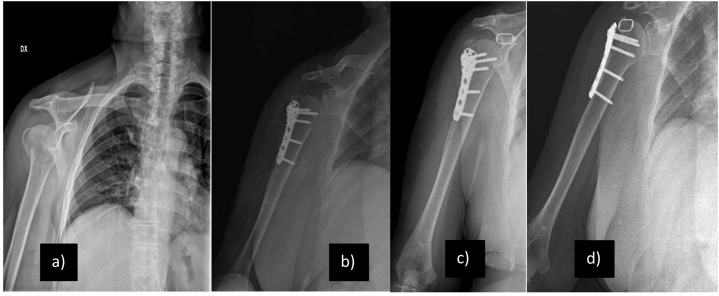
X-ray images of OG patient. a) at the time of trauma; b) post-operative; c) 1 month from operative treatment; d) 6 months from operative treatment. Abbreviations: OG: operative Group.

The OG was treated surgically according to decision-making criteria used by the team of specialists of the Orthopedics and Traumatology Unit, A.O.U. Policlinico-San Marco, University of Catania (Catania, Italy). In this center the surgical technique for proximal humerus fractures treatment mainly includes different options such as intramedullary nails, locking plate or shoulder replacement. For this study were selected only patients treated with non-contact-bridging - NCB ® (Plate for the proximal humerus - PH - Zimmer, Inc.; Warsaw, Indiana, USA) plate fixation. The indications provided by Zimmer, for the use of this plate involves the possibility of inserting it with MIS (Minimally Invasive Solution) approach for the reduction of 2-fragments fractures according to Neer; or in Open technique with Deltoid pectoral incision for 2-, 3-, 4-fragments fractures according to Neer. These patients were treated with MIS approach for all fractures from 1- to 4-fragments according to Neer. (Fig. 3 a-b-*c*-d).

When choosing the type of treatment (conservative vs surgical), attention should be paid to medial comminution, varus angulation, and restoration of the calcar. In addition, other criteria used for taking the decision of surgery were age and functional demands of the patient, possible comorbidities, associated with the ASA physical status classification system risk calculation and the degree of fracture displacement.

The analysis of pain variation (NPRS) leads to a clear reduction of the algic component with both treatment methods. However, starting at T0 from a comparable NPRS value in both treatment groups, from 3 months to one year the reduction of the algic component is certainly greater in patients of the conservative treatment group. This difference could be related to complications associated with the use of plate, such as: intra-articular screw penetration, which was the most reported complication (9.5%), followed by varus collapse (6.8%), subacromial impingement (5.0%), avascular necrosis (4.6%), adhesive capsulitis (4.0%), non-union (1.5%), and deep infection (1.4%). Reoperation occurred at a rate of 13.8%. These data are according to Venkat Kavuri et al. [18] study of the complication of plate fixation. However, in our OG only one patient returned to operating room within first year post treatment.

The score increasing of the Constant-Murley scale is due, as is the case with the Barthel Index, to the reported decrease in pain, improved autonomy in daily activities and increased joint range. Comparing Constant-Murley's age-related scores, it was found that conservatively treated patients presented an average value of 95.2% ± 12.9, instead of patients in the plate fixation group which presented a Constant-Murley's age-related score with an average value of 83.2% ± 18.6. From this we can deduce how the functionality recovery of the fractured limb, in patients examinated, can be considered “good” for the plate's fixations' patients' group and “very good” in patients treated conservatively. In the plate fixation's group, Constatnt-Murley (good) is significantly reduced compared to the one of conservative treatment's group (very good). This could be attributed to the type of surgery used. In fact, plate fixation with MIS technique may not be enough to allow an anatomically perfect reconstruction, unlike plate fixation with deltoid-pectoral access. The MIS technique creates a reduction, improving the fracture condition but with possible imperfections in the restoration the glenohumeral joint surface. We cannot be sure of this hypothesis as part of the study was conducted during the covid-19 lockdown period when it was impossible to call patients into outpatient clinic and perform a new radiographic examination to confirm it. Therefore, to evaluate the outcomes of some patients in the plate fixation group, the scores were obtained by telephone.

In our case history, conservatively treated patients have demonstrated a greater improvement in ROM, on all planes of movement, than patients treated with plate fixation. The use of standardized intensive physio kinesitherapy programs in the conservative treatment group should be considered to better explain the greater improvement of this group. While plate fixation group's patients, physical therapy was prescribed and carried out in another rehabilitation center chosen by the patient and in some cases even at home. In addition, the use of MIS technique for even 3-, 4-fragment fractures may also be responsible for reducing the functional outcome in the plate fixation group. The use of a more expanded access with deltoid pectoral incision with Open technique, as indicated by the Zimmer, could have given a better anatomical reduction and improved functional outcome. So, improving surgical technique could possibly lead to better functional outcomes in patients treated with NCB. Or even the use of other minimally invasive techniques such as the use of the Galaxy external fixator created by Manson in collaboration with Orthofix, that is valid in those cases where the aim is to stabilize the fracture and mobilize the shoulder in the shortest time, especially in elderly patients. Even intramedullary nails and reverse shoulder prostheses can give more effective results in certain fractures.

Study Conclusions of John-Erik Bell et al. [19] was insufficient evidence available to determine whether operative intervention produced better long-term outcomes compared with non-operative management.

But they found that patients undergoing initial open reduction and internal fixation (ORIF) were proportionally more likely to have revision surgery than were patients undergoing initial hemiarthroplasty. Also, Sproul RC et al. [20] in their systematic review of Twelve studies including 514 patients met the inclusion criteria. Analyzing the functional results and complications associated with proximal humerus locking plates, they showed that fixation of proximal humerus fractures with proximal humerus locking plates is associated with a high rate of complications and reoperation.

On the contrary, according to Zyto et al. [21] there is an indication for conservative treatment even in the case of more complex fractures, particularly in patients over 75 years of age.

Fjalestad and colleagues [22] conducted a randomized clinical trial of locked planting versus conservative treatment of 3-, 4- fragments fractures in 50 patients older than 60 years. They found no significative differences in Constant-Murley's score after 1 year of follow up.

K. Okike et al. [23] conducted a comparison of locked plate fixation and nonoperative management in 207 patients over 60 years; in this study the outcomes of plate fixation were like those of conservative treatment, but with higher rate of complications for operative group patients.

These studies compare plate fixation to conservative treatment in subjects over 60 years of age. In our study, the age range of patients included was decreased to 55 years. According to our results, conservative treatment for proximal humerus fractures in patients older than 55 years would appear to be a valid option, according to conclusions obtained by Olerud et al. [24] in their randomized clinical trial of locking plate and non-operative treatment in patients over 55 years. Although outcomes were better in the operative group, differences did not reach statistical significance, and the operative group's reoperation rate was 30%. Also, Rick J. Sanders et al. [25] get similar results to our study. They show that patients treated non-operatively for a proximal humeral fracture achieve better ROM on midterm follow-up (>1 year) compared with patients treated with a locking plate.

The use of plate fixation is widely increasing, especially in elderly patients, even without definitive evidence supporting such management. On the contrary, as mentioned earlier, the literature shows that it may be frequently associated with a higher risk of complications related to the surgical procedure and the following need for reoperation. Hanson et al. [26] analyzed data from 124 patients with proximal humerus fracture (75 to one, 60 to two, 23 to three and 2 to four fragments) treated conservatively (average age 63.3 ± 14.8 years) and completed one year follow-up. It showed that the average Constant Murley score was only 8.2 points different from the healthy limb one year after the acute event and the difference in the average DASH score from the baseline assessment was 10.2 points. The authors concluded that it is difficult to demonstrate a significant advantage of surgical treatment over conservative treatment.

Considering the age above 55 years of the patients in the study, their possible associated comorbidities and the risk of these patients being exposed to a secondary surgery, in according to their residual function demand of the affected limb, it seems that there are no real benefits in the use of plate fixation compared to other minimally invasive techniques or conservative approach.

An important bias in the study that may have affected the results is the age differences between the two groups: CG treated with conservative treatment was composed of 29 patients (5 males, 24 females) with an average age of 70.8 ± 9.9 years; the OG treated with plate fixation was composed of 36 patients (11 males, 25 females) with an average age of 66.2 ± 7.1 years. (p = 0.0331).

Study limitations should be considered: like having considered fracture types as a common group in order to have a more numerous samples, without focusing on a single type; the choice of fracture treatment based on clinical judgement; and being a retrospective study. Limited number of samples (65 patients) due to difficulty of collecting data by telephone evaluation of some patients. This obstacle on collecting people and data was caused by SARS-CoV-2 pandemic period, when calling patients to outpatient clinic was impossible. Short follow-up period that was not adequate for obtaining long-term outcomes. These may have underpowered the study.

Certainly, in the future, data will have to be rechecked, patients will have to be recalled and new clinical and radiographic examinations will have to be carried out. This re-evaluation could redefine the data currently available to us and on which our current considerations are based.

## 5 Conclusions

According to the data resulting from our experience, conservative treatment in proximal humeral fractures, particularly in multi-fragmented fractures in patients over 55, appears to be a very good alternative to the surgical option. Despite the high incidence and costs of proximal humerus fractures, there is currently no valid scientific evidence for the best treatment method [17]. Therefore, the choice of conservative or operational treatment can still be postponed to the critical capacity of the surgeon after a careful analysis of the general clinical codition of the patient and the fracture pattern. And it should have a more prominent role in the treatment of proximal humeral fractures, using surgical treatment in closely selected cases.

## Funding

No funding source to disclose.

## Data availability

Yes.

## CRediT authorship contribution statement

**Marco Sapienza:** Writing – original draft, Validation, Conceptualization. **Vito Pavone:** Writing – review & editing, Supervision, Data curation, Conceptualization. **Liliana Muscarà:** Writing – original draft. **Pierpaolo Panebianco:** Visualization, Software. **Alessia Caldaci:** Data curation. **Kathryn Louise McCracken:** Visualization, Data curation. **Giuseppe Condorelli:** Methodology, Formal analysis, Data curation. **Vincenzo Fabrizio Caruso:** Formal analysis, Data curation. **Danilo Costa:** Writing – original draft, Formal analysis. **Angelo Di Giunta:** Supervision, Methodology, Data curation. **Gianluca Testa:** Writing – review & editing, Supervision, Software, Methodology, Formal analysis, Data curation, Conceptualization.

## Declaration of competing interest

The authors declare the following financial interests/personal relationships which may be considered as potential competing interests: Marco Sapienza reports article publishing charges was provided by University of Catania Faculty School of Medicine. If there are other authors, they declare that they have no known competing financial interests or personal relationships that could have appeared to influence the work reported in this paper.

The authors declare that they have no known competing financial interests or personal relationships that could have appeared to influence the work reported in this paper.
